# Association of cerebrospinal fluid protein biomarkers with outcomes in patients with traumatic and non-traumatic acute brain injury: systematic review of the literature

**DOI:** 10.1186/s13054-021-03698-z

**Published:** 2021-08-05

**Authors:** Carlos A. Santacruz, Jean-Louis Vincent, Andres Bader, Luis A. Rincón-Gutiérrez, Claudia Dominguez-Curell, David Communi, Fabio S. Taccone

**Affiliations:** 1 Department of Intensive Care, Erasme University Hospital, Université Libre de Bruxelles, Route De Lennik 808, 1070 Brussels, Belgium; 2 Department of Intensive and Critical Care Medicine, Academic Hospital Fundación Santa Fe de Bogotá, Bogotá, Colombia; 3 Institut de Recherche Interdisciplinaire en Biologie Humaine Et Moléculaire, Université Libre de Bruxelles, Brussels, Belgium

**Keywords:** Traumatic brain injury, Cerebrospinal fluid, Subarachnoid hemorrhage, Neurological outcomes

## Abstract

**Background:**

Acute brain injuries are associated with high mortality rates and poor long-term functional outcomes. Measurement of cerebrospinal fluid (CSF) biomarkers in patients with acute brain injuries may help elucidate some of the pathophysiological pathways involved in the prognosis of these patients.

**Methods:**

We performed a systematic search and descriptive review using the MEDLINE database and the PubMed interface from inception up to June 29, 2021, to retrieve observational studies in which the relationship between CSF concentrations of protein biomarkers and neurological outcomes was reported in patients with acute brain injury [traumatic brain injury, subarachnoid hemorrhage, acute ischemic stroke, status epilepticus or post-cardiac arrest]. We classified the studies according to whether or not biomarker concentrations were associated with neurological outcomes. The methodological quality of the studies was evaluated using the Newcastle–Ottawa quality assessment scale.

**Results:**

Of the 39 studies that met our criteria, 30 reported that the biomarker concentration was associated with neurological outcome and 9 reported no association. In TBI, increased extracellular concentrations of biomarkers related to neuronal cytoskeletal disruption, apoptosis and inflammation were associated with the severity of acute brain injury, early mortality and worse long-term functional outcome. Reduced concentrations of protein biomarkers related to impaired redox function were associated with increased risk of neurological deficit. In non-traumatic acute brain injury, concentrations of CSF protein biomarkers related to dysregulated inflammation and apoptosis were associated with a greater risk of vasospasm and a larger volume of brain ischemia. There was a high risk of bias across the studies.

**Conclusion:**

In patients with acute brain injury, altered CSF concentrations of protein biomarkers related to cytoskeletal damage, inflammation, apoptosis and oxidative stress may be predictive of worse neurological outcomes.

## Introduction

Acute brain injuries are a group of neurological insults to the brain parenchyma and are associated with poor long-term functional outcomes and high mortality rates [1]. Primary brain injuries represent the initial insult to the brain and are usually considered non-reversible. Secondary brain injuries arise from insults to the brain parenchyma that occur after the initial injury (e.g., as a result of hypoxemia and/or hypotension) and increase the overall area of damaged brain tissue [2, 3]. After an acute brain injury, intrathecal expression of proteins related to brain inflammation, apoptosis and oxidative stress induces production and migration of chemotactic factors, which ultimately lead to blood–brain barrier (BBB) dysfunction, brain edema formation and intracranial hypertension [4]. This cellular response may render the brain more susceptible to secondary injuries in cases of decreased cerebral perfusion pressure and may increase the volume of non-viable tissue.

In humans, the cerebrospinal fluid (CSF) acts as a highly specific repository of cellular by-products, neurotransmitters and protein fragments as it is in close contact with the brain parenchyma and other products of neural origin [5]. Concentrations of protein biomarkers in the intrathecal space may therefore reflect the presence or severity of primary and/or secondary brain injuries. For example, in patients with traumatic brain injury (TBI), increased CSF concentrations of protein biomarkers from damaged neurons may serve as indicators of ongoing cellular damage [6], and, in patients with subarachnoid hemorrhage (SAH), higher concentrations of CSF protein biomarkers may be associated with increased risk of vasospasm and delayed cerebral ischemia [7]. CSF protein biomarkers may reflect the pathophysiological pathways involved in acute brain injuries that could be susceptible to interventions, and thus help in the development of therapies or to guide earlier intervention to improve long-term functional outcomes.

We therefore performed a systematic review to identify observational studies that have evaluated the relationship between CSF protein biomarkers in patients with acute brain injuries and neurological outcomes.

## Materials and methods

### Data sources

Following protocol submission to the Prospero International Prospective Register of Systematic Reviews (ID 114294), we conducted a systematic search of the literature using the MEDLINE database and the PubMed interface from inception until June 29, 2021, to identify all observational studies that evaluated CSF protein biomarkers (proteins were defined as those with at least 50 amino acids or a molecular weight greater than 4000 Da) in patients with severe acute brain injury (as a result of TBI, SAH, acute ischemic stroke, status epilepticus or post-cardiac arrest syndrome) and that reported any neurological outcome. We used the MeSH terms: (((((“Brain Injuries, Traumatic”[MeSH]) OR “Subarachnoid Hemorrhage”[MeSH]) OR “Stroke”[MeSH]) OR “Status Epilepticus”[MeSH]) OR “Post-Cardiac Arrest Syndrome”[MeSH]) AND “Biomarkers”[MeSH]. The search limits were clinical studies, human, adults 19 + (over 18 years of age) and articles written in English. We also searched the references of included articles for studies that had been missed in the initial search. We followed the Preferred Reporting Items for Systematic Reviews and Meta-Analysis (PRISMA) statement [8].

### Study selection and data abstraction

Three of the authors (AB, LARG and CDC) performed the literature search and selected the studies. We excluded studies on descriptive proteomics; those evaluating metabolites (e.g., lactate, lactate/pyruvate, glucose, glutamate, glycerol, etc.), hormones or cytokines/chemokines; those in patients with chronic degenerative or chronic traumatic injuries (e.g., multiple sclerosis, Alzheimer and Parkinson diseases, sport-related injuries, chronic traumatic encephalopathy); those in patients with autoimmune conditions (e.g., Guillain–Barré); pediatric studies; postmortem populations; studies with only physiological outcomes; and animal studies. Data abstraction regarding type of acute brain injury, source of CSF (ventricular or lumbar), number of included subjects, method used by the author to quantify the specific biomarker and neurological outcomes was performed by the same three reviewers (AB, LARG and CDC) in an independent blinded manner by completing predefined tables. Studies were classified according to whether or not the measured biomarker was associated with neurological outcome (as defined in the original study) and were grouped according to whether the brain injury was traumatic or non-traumatic. The methodological quality of the observational studies was evaluated using the Newcastle–Ottawa quality assessment scale [9]. Discrepancies in the assessment of methodologic quality and final classification of the selected studies were resolved by the involvement of a fourth author (CAS).

## Results

**Fig. 1:**
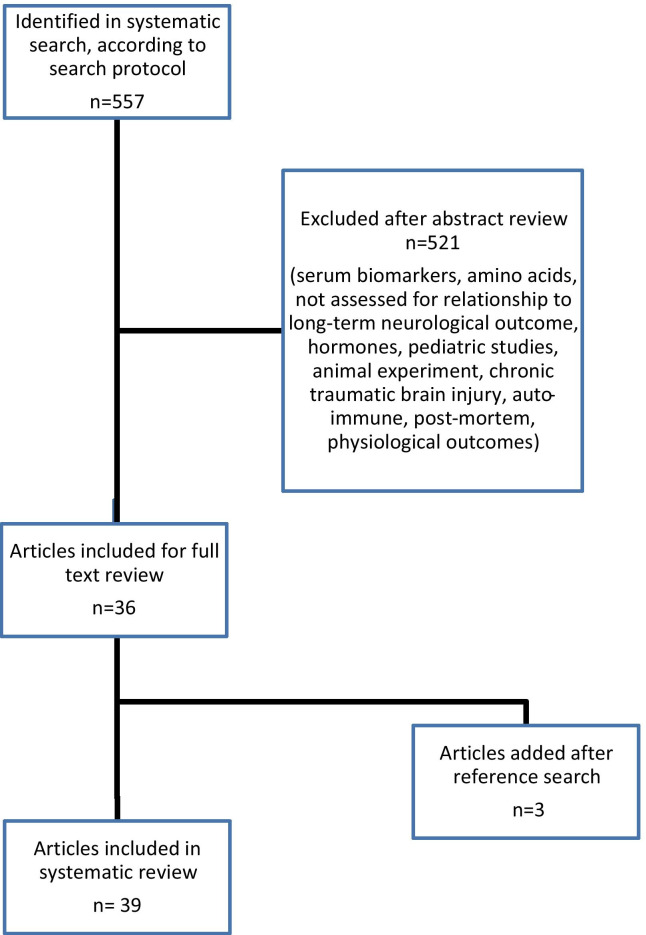
Flowchart of included studies

The initial search yielded 557 citations, and 39 studies met the inclusion criteria (Fig. 1). These studies had evaluated 27 CSF protein biomarkers; 26 studies had evaluated the relationship of a protein biomarker in acute brain injuries of traumatic origin [10, 12, 15, 17–22, 24–29, 32, 34, 36, 39, 41–47], 11 in acute brain injuries of non-traumatic origin [7, 11, 13, 16, 23, 30, 31, 33, 35, 37, 38] and two in acute brain injuries of mixed (traumatic and non-traumatic) origin [14, 40]. No study had reported CSF biomarkers after cardiac arrest. Thirty studies [7, 10–38] reported an association of the protein biomarker with neurological outcome (Table 1), and 9 reported no association [39–47] (Table 2).

**Table 1:** Trials in which cerebrospinal fluid (CSF) protein biomarkers were associated with neurological outcome

Author (ref)	Study population	CSF biomarker	Biological function of biomarker	Number of patients (ABI/control)	Source of protein (ABI/control)	Time point of first sampling*	Method of biomarker detection	Outcome measure	Relationship of biomarker with outcome	Newcastle–Ottawa risk of bias
Jiang et al. 2020 [10]	TBI	Caspase-3, cytochrome C, sFas and caspase-9	Apoptosis	45/25	vCSF/lCSF	Day 1 after injury	ELISA	6-month GOS	ICP and caspase-3 were significant predictors of outcome at 6 months	★★★★★
Mertens et al. 2018 [11]	Ischemic	Procarboxypeptidase U (proCPU, TAFI, proCPB2)	Inflammation, coagulation	AIS (n = 58) or TIA (n = 14)/32	lCSF/lCSF	Day 1 after symptoms onset	ELISA	3-month mRS	Increased proCPU levels were associated with stroke progression and worst mRS	★★★★
Kerr et al. 2018 [12]	TBI	Caspase-1, apoptosis-associated speck-like protein containing a caspase recruitment domain (ASC)	Apoptosis	21#/30	NS/Biobank	Day 1 after injury	ELISA	GOSE	Higher protein levels of ASC were consistent with poorer outcomes after TBI	★★★★★
Wąsik et al. 2017 [13]	SAH	Clusterin	Apoptosis	27/25	vCSF/lCSF	Day 1 after bleeding	ELISA	3- month GOS	Higher levels of CSF clusterin were found 5–7 days after SAH in patients with good outcome	★★★★
Kellermann et al. 2016 [14]	Mixed	S-100β	Cytoskeleton	45 SAH—57 TBI/no control	vCSF/no control	Day 1 after EVD placement	ELISA	GOS	In TBI and SAH patients, S-100β concentrations in CSF and serum were significantly higher in patients with unfavorable outcome (GOS 1–3)	★★
Failla et al. 2016 [15]	TBI	BDNF	Cytoskeleton	203/10	vCSF and serum/lCSF and serum	NR	ELISA	1-year mortality	Higher CSF levels predicted mortality	★★★★★★
Wu et al. 2016 [16]	SAH	NLRP1, ASC and caspase 1	Apoptosis	24/10	vCSF-lCSF/lCSF	Between 24 and 72 h after injury	SDS-PAGE	3-month GOS	Higher levels of inflammasome proteins were associated with severe SAH and poor outcome at 3 months	★★★
Papa et al. 2015 [17]	TBI	UCH-L1, MAP-2, SBDP150, SBDP145, SBDP120, MBP and S-100β	Apoptosis, cytoskeleton	131/21	vCSF/mix^b^	6 h after injury	ELISA	6-month mortality	MAP-2 in combination with clinical data provide enhanced prognostic capabilities for mortality at 6 months	★★★
Manevich et al. 2014 [18]	TBI	Peroxiredoxin (Prdx) VI	Redox	21/10	vCSF/lCSF	During EVD placement after injury	Western Blot	Scale of neurological deficits at discharge	Reduction of Prdx appeared to correlate with milder neurological deficits	★★★
Liu et al. 2014 [19]	TBI	Matrix metalloproteins (MMP-9)	Inflammation	6/85	vCSF/vCSF	During EVD placement	ELISA	ICP and GCS	MMP-9 was negatively correlated with the Glasgow Coma Scale	★★★
Gatson et al. 2013 [20]	TBI	NSE and Ab42	Energy, neurodegeneration	18/no control	vCSF/no control	Within 72 h after injury	ELISA	GOS-E and DRS	CSF oligomer levels correlated with GOS-E scores	★★
Mondello et al. 2013 [21]	TBI	Alpha-synuclein	Neurodegeneration	12/22	vCSF/lCSF	NR	ELISA	6-month GOS-E Mortality	Rising levels predicted mortality with 100% specificity and high sensitivity (83%)	★★★
Goyal et al. 2013 [22]	TBI	S100β	Cytoskeleton	138/15	vCSF /lCSF	First 6 days post-injury	ELISA	GOS DRS Mortality	Mean and peak levels were associated with mortality and GOS scores, but not with DRS	★★★★★
Zanier et al. 2013 [23]	SAH	H-FABP and tau protein	Cytoskeleton	38/16	vCSF/lCSF	Day 1 after injury	ELISA	GOS	Higher H-FABP and tau levels in patients with unfavorable outcome (death, vegetative state or severe disability)	★★★★★
Adamczak et al. 2012 [24]	TBI	ASC, caspase-1 and NALP-1	Apoptosis	23/9	vCSF/vCSF	Within 12 h of injury and up to 72 h after injury	Western Blot	5-month GOS	Expression of each protein correlated significantly with the GOS at 5 months post-injury	★★★
Böhmer et al. 2011 [25]	TBI	NSE, S-100β and glial fibrillary acidic protein	Cytoskeleton	20/20	vCSF/lCSF	Between 2 and 4 h after hospitalization	ELISA	Survival	At admission, CSF NSE level predicted brain death more accurately than S-100β	★★★★★★
Stein et al. 2011 [26]	TBI	S100β, NSE	Cytoskeleton	23/no control	vCSF/no control	Upon insertion of the EVD or as soon as possible after consent was obtained	ELISA	ICH CH	S-100β and NSE levels were associated with ICH and CH	★★
Darwish et al. 2010 [27]	TBI	Cytochrome c and activated caspase-9	Apoptosis	9/5	vCSF/lCSF	2 to 6 h after injury	ELISA	GOS	Activated caspase-9 showed weak correlation with poor neurologic outcome	★★★
Mondello et al. 2010 [28]	TBI	SBDP145- SBDP120	Apoptosis	40/24	vCSF/vCSF	First 24 h after injury	ELISA	3-month survival	CSF SBDP levels predicted injury severity and mortality after severe TBI	★★★
Papa et al. 2010 [29]	TBI	UCH-L1	Neurodegeneration	41/25	vCSF/vCSF	6 h after injury	ELISA	GOS, 6-week mortality	Higher levels in patients with lower GCS score at 24 h, in those with post-injury complications, in those with 6-wk mortality and in those with a poor 6-month dichotomized GOS	★★★★★
Brouns et al. 2010 [30]	Ischemic	MBP, GFAP, S100β, NSE	Cytoskeleton, energy	89/35	lCSF/ lCSF	NR	ELISA	3-month mRS Infarct volume	MBP was a marker for infarct location. GFAP and S-100β correlated with stroke severity and outcome	★★★
Fountas et al. 2009 [31]	SAH	CRP	Inflammation	41/no control	vCSF	Admission	Nephelometry	GOS, mRS	Increased CRP in CSF associated with increased risk of vasospasm and bad outcome	★★★
Pineda et al. 2007 [32]	TBI	SBDP	Apoptosis	41/11	vCSF/vCSF	6 h after injury	SDS-PAGE	6-month GOS, severity of injury, computed tomography (CT) scan findings	SBDP correlated with severity of injury, computed tomography (CT) scan findings and outcome at 6 months post-injury	★★★
Lewis et al. 2007 [33]	SAH	α-2 spectrin and SBDP	Apoptosis	20/10	vCSF /lCSF	NR	SDS-PAGE	6-month GOS, vasospasm	SBDP levels were significantly increased in patients with vasospasm	★★★★
Ost et al. 2006 [34]	TBI	c-tau	Cytoskeleton	39/20	vCSF /lCSF	First 24 h after injury	ELISA	GOSE	vCSF total tau on days 2 to 3 post-trauma correlated to morbidity and mortality at 1 year	★★★
Selakovic et al. 2005 [35]	Ischemic	NSE	Energy	55/16	lCSF/ lCSF	1–2 days [21 patients], 3–4 days [14 patients], and 5–7 days [20 patients] from the onset of symptoms	ELISA	Infarct volume, Canadian neurological scale and Barthel index	Significant correlation between NSE concentration and infarct volume and degree of neurological and functional deficit	★★★
Kay et al. 2003 [7]	SAH	Apo-E and S-100β	Inflammation, cytoskeleton	19/28	vCSF/lCSF	Within 72 h after injury	ELISA	3-month GOS	SAH patients with more severe injury and less favorable outcome had lower CSF apo-E concentration	★★★
Zemlan et al. 2002 [36]	TBI	C-tau	Cytoskeleton	28/154	vCSF/ lCSF	NR	ELISA Immunoblotting	GOS	C-tau levels-independent predictor of clinical outcome	★★★★
Aurell et al. 1991 [37]	Ischemic	S-100β and glial fibrillary acidic protein	Cytoskeleton	28/18	lCSF/lCSF	12–48 h after onset of symptoms	ELISA (S-100β) Radioimmunoassay (GFAP)	Clinical state: Simplified activities of daily living test Size of infarct: computed tomography	Increment was significantly correlated with size of infarction and clinical state of patients	★★★★★
Strand et al. 1984 [38]	Ischemic	MBP, tau-fraction, albumin, IgG and transferrin	Cytoskeleton, inflammation	40/37	lCSF/lCSF	24 h after symptoms onset	Radioimmunoassay (MBP); crossed immunoelectrophoretic method (tau-fraction); electroimmunoassay (albumin, IgG and transferrin)	Disability groups, mortality	MBP increased with extent of brain injury; high values indicated poor short-term prognosis for the patient. No clear patterns for other markers	★★★★★

*ABI* acute brain injury, *AIS* acute ischemic stroke, *Apo-E* apolipoprotein E, *ASC* apoptosis-associated speck-like protein containing a caspase recruitment domain, *BDNF* brain-derived neurotrophic factor, *CRP* C-reactive protein, *lCSF* lumbar CSF, *C-tau* cleaved tau protein, *DRS* Disability Rating Scale, *ELISA* enzyme-linked immunosorbent assay, *EVD* external ventricular drainage,  *GCS* Glasgow Coma Score, *GOS* Glasgow Outcome Scale*, GOS-E* extended Glasgow Outcome Scale, *H-FABP* heart-type fatty acid binding protein, *ICP* intracranial pressure, *MAP-2* microtubule-associated protein, *mRS* modified Rankin Scale, *MBP* myelin basic protein, *MMP* matrix metalloproteinase, *NALP1* nacht leucine-rich-repeat protein-1, *NR* not reported, *NSE* neuron-specific enolase, *UCH-L1* ubiquitin C-terminal hydrolase, *SDS-PAGE* sodium dodecyl sulfate polyacrylamide gel electrophoresis, *S-100β* S-100 beta, *SBDP* spectrin breakdown products, *TAFI* thrombin-activatable fibrinolysis inhibitor, *TBI* traumatic brain injury, *TIA* transient ischemic attack, *vCSF* ventricular CSF

^#^18 CSF samples; *as reported by the author

**Table 2:** Trials where cerebrospinal fluid (CSF) protein biomarkers were not associated with neurological outcome

Author (ref)	Study population	Biomarker	Biological function of biomarker	Number of patients (ABI/control)	Source of protein (ABI/control)	Time point of first sampling*	Method of biomarker detection	Outcome measure	Quality (Newcastle–Ottawa)
Jha et al. 2017 [39]	TBI	Sulfonylurea receptor-1	Energy	28/15	vCSF/biobank	24 h after injury	ELISA	3-month GOS^a^	★★★
Martinez-Morillo et al. 2015 [40]	Mixed	NMP	Cytoskeleton	30 HS, 11 IS/10	Biobank/biobank	Median 5 (0–9) days in HS group and 1 (0–3) day in IS group	ELISA	3-month GOS	★★★★★
Bellander et al. 2011 [41]	TBI	S-100β	Cytoskeleton	20/no control	vCSF/no control	At admission	Chemiluminometric immunoassays	3–12-month GOS	★★
Grossetete et al. 2009 [42]	TBI	MMP-2 and MMP-9	Inflammation	6/4	vCSF/vCSF	Following EVD insertion	Gelatin zymography and Western Blot	RLAFS and GOS	★★★
Cardali et al. 2006 [43]	TBI	α-2 spectrin and SBDP	Apoptosis	8/2	vCSF/vCSF	6 h after injury	Western blot and SDS-PAGE	GOS	★★★
Farkas et al. 2005 [44]	TBI	Spectrin and SBDP	Apoptosis	12/14	vCSF/mix	Following EVD insertion	ELISA	GOS	★★★
Kay et al. 2003 [45]	TBI	Apo E + S100β	Inflammation, cytoskeleton	27/28	vCSF/lCSF	Within three days post-injury	ELISA	GOS	★★★
Franz et al. 2003 [46]	TBI	Aβ-amyloid 1–42 and tau protein	Neurodegeneration, cytoskeleton	29/31	15 vCSF, 14 lCSF/lCSF	Between 1- and 284-days post-injury	ELISA	GOS	★★★
Raby et al. 1998 [47]	TBI	β-amyloid peptide 1–42	Neurodegeneration	6/24	vCSF/vCSF	NR	ELISA Western blot	GOS	★★★★

*ABI* acute brain injury, *Apo-E* apolipoprotein E, *vCSF* ventricular CSF, *lCSF* lumbar CSF, *C-tau* cleaved tau protein, *DRS* Disability Rating Scale, *ELISA* enzyme-linked immunosorbent assay, *GOS* Glasgow Outcome Scale, *HS* hemorrhagic stroke, *IS* ischemic stroke, *NR* not reported, *MMP* matrix metalloproteinase, *NMP* neurofilament medium polypeptide, *SDS-PAGE* sodium dodecyl sulfate polyacrylamide gel electrophoresis, *RLAFS* Rancho Los Amigos functional scale, *S-100Β* S-100 beta, *SBDP* spectrin breakdown products, *TBI* traumatic brain injury; *HS* hemorrhagic stroke, *IS* ischemic stroke

*As reported by the author

^a^mean and peak sulfonylurea receptor-1 were elevated in patients with CT edema

### Observational trials reporting biomarker associations with neurological outcome

Of the 30 trials that reported a biomarker associated with outcome, 18 included patients with TBI (n = 1345), 6 included patients with SAH (n = 258), 5 included patients with acute ischemic stroke (n = 422), and one included a mixed population (TBI and SAH) (n = 102). The main biological functions reflected by the biomarkers were related to primary brain injury (neuron cell cytoskeleton) and secondary brain injury, e.g., increased apoptosis, inflammation and energy metabolism, reduced redox response to oxidative stress and increased neurodegeneration. Specifically, concentrations of the CSF biomarkers ubiquitin carboxy-terminal hydrolase L1 (UCH-L1), microtubule-associated protein (MAP)-2, alpha-synuclein and peroxiredoxin VI were associated with a lower Glasgow Coma Scale (GCS) score on admission, worse long-term functional outcome and increased mortality. In patients with SAH, NLRP1, ASC (apoptosis-associated speck-like protein containing a caspase recruitment domain), caspase-1 and 3, α-2 spectrin and SBDP (spectrin breakdown products), apolipoprotein-E, S-100β, H-FABP (heart-type fatty acid binding protein) and tau protein were associated with an increased risk of vasospasm, late cerebral ischemia and worse functional outcome at 3–6 months. These findings were consistent when the CSF was collected from a mixed cohort of TBI and SAH patients. In patients with acute ischemic stroke, proteins related to cytoskeleton disruption and energy metabolism were consistently associated with the size of brain infarction and clinical status (see Table 1).

### Observational trials reporting no association of the biomarker with neurological outcome

Of the 9 trials that reported no association of the biomarker with neurological outcome [39–47], 8 included patients with TBI (n = 254) and 1 had a mixed population of patients with hemorrhagic or ischemic stroke (n = 51). The main biological functions assessed by the studied biomarkers included inflammation, neuronal cytoskeleton components, apoptosis, energy metabolism and neurodegeneration (Table 2).

### Methodological analysis

The risk of bias among the included studies was high according to the Newcastle–Ottawa scale [9] (Tables 1 and 2). In addition, different CSF sources were used for assessment of protein biomarker concentrations (ventricular CSF, lumbar CSF, serum, biobanks) across different studies and most control group patients also had neurological conditions that may have influenced biomarker concentrations (e.g., normal pressure hydrocephalus). The studies of patients with acute ischemic stroke were the only ones in which the source of CSF was always the same in the intervention and the control group (lumbar CSF).

## Discussion

Our results suggest that CSF concentrations of protein biomarkers associated with the pathophysiological pathways involved in acute brain injuries may be predictive of increased morbidity and mortality after traumatic and non-traumatic acute brain injury.

CSF proteomic expression may be altered by many factors including genetic background, the severity of the primary brain injury and secondary insults, such as hypoxemia and hypotension [48, 49]. In patients with a traumatic origin of the acute brain injury, cytoskeletal damage was associated with an increased risk of cerebral hemorrhage, intracranial hypertension and early mortality rates, suggesting severe primary brain injuries. After the initial phase of acute brain injury, the expression of proteins involved in re-establishing normal homeostasis is altered [50]. If this response is dysregulated, it may overwhelm counter-regulatory measures initiated by the body to reduce tissue injury, increasing the risk of secondary brain injuries [51]. Moreover, impairment of normal biological functions (e.g., redox function capability, dysregulated inflammation, increased apoptosis) after a primary acute brain injury may render the brain more susceptible to secondary injuries. This seems to be the case in patients with SAH in whom CSF concentrations of C-reactive protein [31], α-2 spectrin and SBDP [33], apolipoprotein E [7], H-FABP and tau protein [23] were associated with an elevated risk of vasospasm and delayed cerebral ischemia. Interestingly, in a mixed population of patients with traumatic and non-traumatic acute brain injuries, concentrations of the structural protein S-100β were higher in patients with lower Glasgow Outcome Scale (GOS) scores [14], suggesting a common pathophysiological pathway for these two types of injury.

Consequences such as acute brain edema, vasospasm or non-convulsive status epilepticus are of crucial importance in patients with acute brain injury because they may affect long- and short-term outcomes. Jha et al. [39] evaluated the ability of the protein biomarker sulfonylurea receptor-1 (Sur1) to predict the risk of cerebral edema in patients with severe TBI. Patients with evidence of edema on computed tomography (CT) had higher concentrations of Sur1 with statistically significant differences in mean (*p* = 0.023) and peak (*p* = 0.019) concentrations in patients with and without edema. Although there were no differences in functional outcome, as assessed using the 3-month GOS score, in patients with higher Sur1 concentrations, prediction of cerebral edema may indicate the need for more aggressive therapeutic measures.

It is difficult to imagine that a single biomarker could explain the complex cascades of events following acute brain injury that may be related to worse long-term outcomes. A single CSF protein biomarker may indicate derangement of a specific biological function but may not be involved in other pathophysiological pathways. Moreover, the time point at which the biomarker is measured may reflect different stages of acute brain injury (e.g., primary vs secondary injury). Thus, earlier sampling of CSF biomarkers after initial injury may provide information about the severity of the initial injury (e.g., increased risk of early mortality, extent of brain tissue involvement, risk of severe intracranial pressure), whereas more delayed measurements could provide information on risk of chronic degenerative encephalopathy or longer-term outcomes. This could be an interesting area for future study.

Our review has several limitations. First, the search strategy was based solely on the MEDLINE database, and more studies may have been identified if other databases (e.g., Embase) had been used. Second, because of insufficient data we could only provide descriptive data. We were unable to determine which protein biomarker was most associated with worse short- or long-term outcomes. Also, there was a high risk of bias among the included studies because of trials without a control group, a control group with CSF-derived from patients with other neurological conditions (e.g., with normal pressure hydrocephalus) or studies comparing lumbar and ventricular CSF without taking into account the craniocaudal gradient [52]. Finally, some studies used frozen biobank samples, which may have lower protein concentrations because of proteolysis induced by freeze–thaw and contamination. Future studies should report in a more standardized fashion to enable comparison across different studies.

## Conclusions

Changes to the CSF proteome in patients with acute brain injury reflecting the pathophysiological pathways involved may be indicative of the severity of the injury and predictive of worse neurological outcomes. However, there are currently insufficient data available to recommend the routine measurement of any CSF biomarker in these patients.

## Data Availability

Not applicable.

## Abbreviations

ABI: Acute brain injury
Apo-E: Apolipoprotein E
CSF: Cerebrospinal fluid
C-tau: Cleaved tau protein
DRS: Disability Rating Scale
ELISA: Enzyme-linked immunosorbent assay
GOS: Glasgow Outcome Scale
GOS-E: Extended Glasgow Outcome Scale
H-FABP: Heart-type fatty acid binding protein
MBP: Myelin basic protein
MMP: Matrix metalloproteinase
NMP: Neurofilament medium polypeptide
NSE: Neuron-specific enolase
RLAFS: Rancho Los Amigos functional scale
SAH: Subarachnoid hemorrhage
SBDP: Spectrin breakdown products
SDS-PAGE: Sodium dodecyl sulfate polyacrylamide gel electrophoresis
Sur1: Sulfonylurea receptor-1
TBI: Traumatic brain injury
UCH-L1: Ubiquitin C-terminal hydrolase
